# Kinetochore–microtubule error correction for biorientation: lessons from yeast

**DOI:** 10.1042/BST20221261

**Published:** 2024-02-02

**Authors:** Shuyu Li, Taciana Kasciukovic, Tomoyuki U. Tanaka

**Affiliations:** Division of Molecular, Cell and Developmental Biology, School of Life Sciences, University of Dundee, Dow Street, Dundee DD1 5EH, U.K.

**Keywords:** budding yeast, chromosome biorientation, chromosome segregation, error correction, kinetochore, microtubule

## Abstract

Accurate chromosome segregation in mitosis relies on sister kinetochores forming stable attachments to microtubules (MTs) extending from opposite spindle poles and establishing biorientation. To achieve this, erroneous kinetochore–MT interactions must be resolved through a process called error correction, which dissolves improper kinetochore–MT attachment and allows new interactions until biorientation is achieved. The Aurora B kinase plays key roles in driving error correction by phosphorylating Dam1 and Ndc80 complexes, while Mps1 kinase, Stu2 MT polymerase and phosphatases also regulate this process. Once biorientation is formed, tension is applied to kinetochore–MT interaction, stabilizing it. In this review article, we discuss the mechanisms of kinetochore–MT interaction, error correction and biorientation. We focus mainly on recent insights from budding yeast, where the attachment of a single MT to a single kinetochore during biorientation simplifies the analysis of error correction mechanisms.

## Introduction

Chromosomes contain vital genetic information. So, to maintain genetic integrity, proper chromosome segregation is required during mitosis in eukaryotic cells, i.e. duplicated chromosomes (sister chromatids) should separate accurately and be distributed equally between two daughter cells. Correct and stable interaction between the kinetochore and microtubules (MTs) is essential for high-fidelity chromosome segregation. The yeast kinetochore is a large protein complex, comprising over 50 components, and organized into distinct subcomplexes such as COMA, MIND and the Ndc80 complex (Ndc80C) [1,2], all of which assemble at the point centromere region in the budding yeast [3]. Despite vast differences in centromeric DNA across species, the fundamental architecture of the kinetochore is conserved from yeast to humans [4]. Budding yeast serves as an excellent model system for studying kinetochore–MT interaction not only because of the versatile molecular genetics in this organism but also because of each kinetochore binding to a single MT [5], simplifying the analysis of this process.

**Figure 1.**
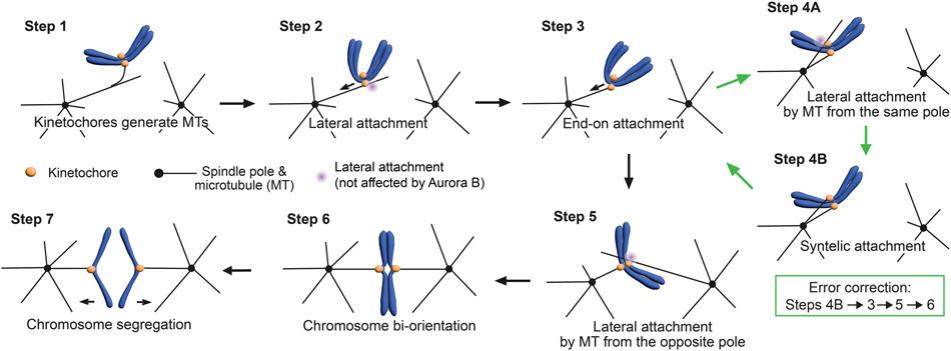
The diagram shows kinetochore–MT interaction during mitosis. Each step of the process is detailed in the text. The transition from Steps 4B, 3, 5 to 6 represents the error correction. Note that Steps 1, 2 and 3 occur before the separation of the spindle poles (establishment of the bipolar spindle) in budding yeast [23], but these steps (and other steps) occur after it in vertebrate cells (as shown here).

In yeast, MTs extend from spindle poles that are organized by spindle pole bodies (SPBs). MTs have dynamic, hollow, tube-like structures, and they undergo phases of growth and shrinkage through the addition and removal of tubulin heterodimers at their plus ends. Kinetochore–MT interaction occurs in a stepwise manner: Initially, the kinetochore attaches to the lateral side of an MT extending from a spindle pole (lateral attachment), which is a conserved step from yeast to vertebrates [6,7]. The MT lateral side provides a large surface for kinetochore interaction, and the lateral attachment is often assisted by a transiently formed kinetochore-derived short MT that interacts with an MT extending from a spindle pole (Figure 1, Step 1) [8–11]. Subsequently, kinetochores move along the MT towards the spindle pole, which is driven by Kar3 (kinesin 14) motor in budding yeast and the dynein motor in animal cells (Step 2) [7,12,13]. The lateral attachment transitions to the end-on attachment where the kinetochore attaches to the MT plus end as the MT depolymerizes and its plus end interacts with the kinetochore (Step 3) [14,15]. If sister kinetochores aberrantly attach to MTs from the same pole (syntelic attachment, Step 4B), at least one of the kinetochore–MT interactions should be removed to allow a fresh interaction (Figure 1, error correction) [16,17]. Once sister kinetochores attach to MTs extending from opposite poles (biorientation establishment), tension is applied across sister kinetochores, stabilizing kinetochore–MT interactions (Steps 5, 6) [18].

Chromosome biorientation is fundamental for correct chromosome segregation in anaphase, and error correction is crucial for the establishment of biorientation. Although several mechanisms for the regulation of error correction have been identified or suggested, the precise dynamics for the exchange (or turnover) of kinetochore–MT interactions during error correction remain elusive. It is also not clear how tension stops this exchange and stabilizes kinetochore–MT interaction. In this review article, we discuss how the study of budding yeast, *Saccharomyces cerevisiae*, as a model organism has helped our understanding of error correction mechanisms. We highlight research advancements made over the last 5 years. It is worth noting that many regulators, discussed here, are also involved in the spindle-assembly checkpoint (SAC). However, in this review, we do not discuss the SAC that was covered in other recent reviews [19,20].

## Kinetochore–microtubule interface in budding yeast: the Ndc80 and Dam1 complexes and their regulation

The kinetochore–MT interface in budding yeast is organized through the concerted action of the outer kinetochore complexes and MT-associated proteins and motors. The Ndc80C and the Dam1 complex (Dam1C, also called DASH) are major outer kinetochore components in budding yeast, which directly interact with MTs and play major roles in forming kinetochore–MT interface [1,21,22]. The Ndc80C, consisting of four proteins (Ndc80, Nuf2, Spc24, and Spc25), is a conserved outer kinetochore protein complex that plays a crucial role in both the lateral and end-on attachment of kinetochores to spindle MTs [7]. The Dam1C, which consists of 10 proteins including Dam1, Duo1, Ask1, Spc34 and Spc19, is not part of the kinetochore and localizes at the MT plus end during the lateral attachment, but subsequently interacts with Ndc80C to provide a kinetochore–MT interface for the end-on attachment [15,17,23]. The Dam1Cs oligomerizes into a partial or complete ring encircling an MT *in vitro* [24,25], and couples the kinetochore motion to the MT plus end [26–28]. To resolve aberrant kinetochore–MT interactions, Aurora B kinase (Ipl1 in budding yeast) phosphorylates the Dam1C components and Ndc80 protein to weaken and disrupt the end-on attachment [29–31]. The phosphorylation of Dam1C is fundamental in this process, while the phosphorylation of the Ndc80 N-terminus modestly contributes to it by weakening Ndc80C–MT interaction. New functional and structural information on Ndc80C and Dam1C have been obtained in recent years, thanks to advancements in biochemical reconstitution, cryo-electron microscopy (cryo-EM) technique, AI-based structure prediction (AlphaFold2) and super-resolution microscopy.

Although the Dam1Cs form a partial or complete ring around an MT *in vitro* [24,25], it had been unclear whether this is also the case *in vivo* (i.e. within cells). Using electron tomography reconstitution of *in situ* serial cryosections in budding yeast cells, it was recently shown that Dam1Cs oligomerize into a partial or complete ring around an MT in the vicinity of the MT plus ends *in vivo* during metaphase [32]. Moreover, within the ring, each Dam1C forms a ‘bridge’ that directly interacts with the MT wall both *in vitro* and *in vivo* [32]. Another study showed that MT-associated protein Bim1 stably binds a Dam1C component Duo1 and promotes the assembly of the Dam1C ring around an MT [33]. In addition, phosphorylation of a Dam1C subunit, Ask1, by Cdk1 kinase was also suggested to enhance the Dam1C ring assembly (or Dam1C affinity to an MT) and strengthen the kinetochore–MT interaction [34]. Moreover, the intermediate filament protein Fin1 localizes at the kinetochore and adjusts the stoichiometry of Ndc80C and Dam1C in anaphase [35]. Furthermore, using nanoscale super-resolution microscopy (single-molecule localization microscopy), individual kinetochores were distinctly visualized *in vivo* in the yeast mitosis [36]. This demonstrated not only the precise location and distribution of individual kinetochores in the nucleus but also the relative positions and copy numbers of kinetochore components (including Ndc80C and Dam1C) within each kinetochore [36].

For the establishment of biorientation, phosphorylation of the following Dam1C components by Aurora B is crucial for error correction: Dam1 (S20, S257, S265 and S292), Ask1 (S200) and Spc34 (T199), where their residues for phosphorylation are shown in parentheses [30]. Recent studies addressed how these phosphorylated residues disrupt end-on attachment to promote error correction. Evidence suggested that the phosphorylation of Dam1 S20 is important to reduce oligomerization (or ring formation) and MT binding of Dam1Cs [27,37], while the clustered phosphorylation at Dam1 C-terminus (S257, S265 and S292) is crucial to weaken the Dam1 interaction with the hairpin region of Ndc80 [17,38–41]. Moreover, using protein cross-linking and mass spectrometry it was shown that Dam1 C-terminus, Ask1 middle region (including S200) and Spc34 C-terminus (including T199) interact with three different regions of Ndc80C [42]. It was also suggested that phosphorylation of Ask1 S200 and Spc34 T199 contributes to weakening the Dam1C–Ndc80C interaction [42].

**Figure 2.**
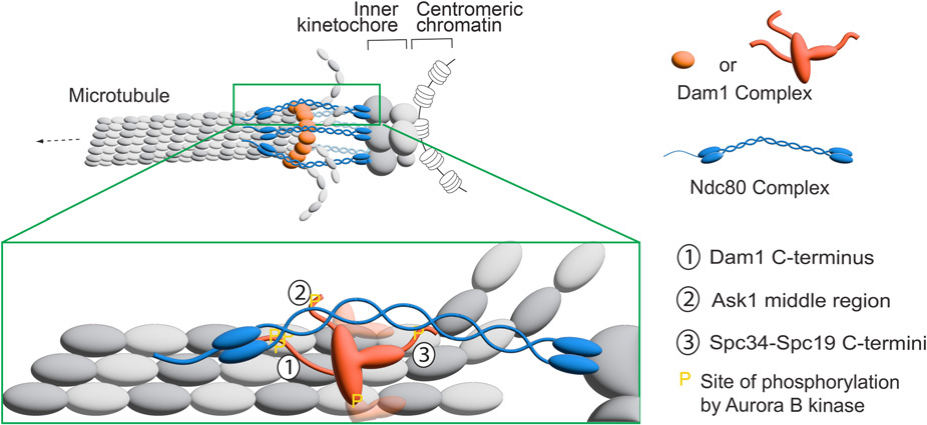
The interaction between the Dam1 and Ndc80 complexes at the kinetochore–MT interface. The diagram shows the interaction between Dam1C and Ndc80C during the end-on attachment in budding yeast based on [42,43]. The Dam1 C-terminus, Ask1 middle region and Spc34/Spc19 C-termini interact with different regions of the Ndc80C. Sites of phosphorylation on Dam1C by Aurora B kinase [30] are shown by ‘P’ in yellow.

Meanwhile, the detailed structure of the core Dam1C ring was revealed using cryo-EM [43]. Although the structure did not include the Dam1 C-terminus and Ask1 middle region as they are flexible, a structural model was proposed for how these regions and Spc34 C-terminus interact with the three different regions of Ndc80C [43] (Figure 2). Subsequently, it was shown that the three interactions between Dam1C and Ndc80C were weakened by phosphorylation of Dam1, Ask1 and Spc34 by Aurora B (with the effect being greater in this order) [44]. Most recently, cryo-EM and AlphaFold2 studies demonstrated how phosphorylation of Dam1, Ask1 and Spc34 by Aurora B can weaken the end-on attachment. The Dam1 N-terminus localizes at the interface between individual Dam1Cs in their oligomerized form. S20 of Dam1 is buried at this interface, and it was suggested that S20 phosphorylation would disrupt this interface, thus explaining how this phosphorylation inhibits Dam1C oligomerization and ring formation [45]. Moreover, two short segments of Dam1 residues 251–272 and 287–301 interact with the Ndc80–Nuf2 coiled-coil next to their calponin-homology (CH) domains, and it was suggested that phosphorylation of S257, S265 and S292 would disrupt these interactions [45,46]. The structure also demonstrated how the Spc34 C-terminus (including T199) interacts with the central segment of the Ndc80–Nuf2 coiled-coil [45]. Moreover, the identified Dam1C–Dam1C and Dam1C–Ndc80C interaction sites were indeed important for cell viability and for withstanding forces in an optical tweezer assay [45]. Thus, recent works demonstrated the structural details regarding (1) how the Dam1Cs oligomerize and form a ring encircling an MT and (2) how Dam1Cs interact with Ndc80Cs. These works also suggest how phosphorylation of Dam1, Ask1 and Spc34 by Aurora B disrupts the Dam1C ring formation and Ndc80C–Dam1C interactions through steric hindrance and charge repulsion to promote error correction.

## Kinetochore–microtubule interactions exchange during error correction

As discussed in section “Kinetochore–microtubule interface in budding yeast: the Ndc80 and Dam1 complexes and their regulation”, aberrant kinetochore–MT interaction is disrupted through phosphorylation of Dam1C and Ndc80C by Aurora B kinase. This process is sensitive to tension applied to kinetochore–MT interaction, i.e. under low tension Dam1C phosphorylation occurs, promoting error correction [47,48]. In contrast, when biorientation is established and high tension is applied, Dam1C is dephosphorylated and kinetochore–MT interaction is stabilized [47,48]. To establish biorientation, a new kinetochore–MT interaction must be formed after the aberrant one is removed. This may constitute a conundrum since the new interaction should be discouraged by Aurora B while tension is low. However, it was shown the kinetochore interaction with the MT lateral side (lateral attachment) is impervious to the action of Aurora B, while the kinetochore interaction with the MT end (end-on attachment) is disrupted by Aurora B under low tension [17]. It was, therefore, suggested that, after aberrant end-on attachment (in which both sister kinetochores interact with MTs from the same spindle pole) is resolved (Figure 1, Steps 4B–3), a new lateral attachment is formed. If this results in an aberrant attachment (Figure 1, Step 4A), its resolution is promoted by Aurora B again. However, if biorientation is established, tension is applied and kinetochore–MT interaction is stabilized (Figure 1, Steps 5, 6) [17]. The differential regulation of lateral and end-on attachments by Aurora B may occur since (1) Dam1C is the main Aurora B substrate for error correction and (2) Dam1C is involved in the end-on, but not lateral, kinetochore attachment (see section “Kinetochore–microtubule interface in budding yeast: the Ndc80 and Dam1 complexes and their regulation”). Since phosphorylation of Dam1C components by Aurora B regulates Dam1C–Dam1C and Dam1C–Ndc80C interactions (see section “Kinetochore–microtubule interface in budding yeast: the Ndc80 and Dam1 complexes and their regulation”), Dam1C, Ndc80C and Aurora B may be sufficient to recapitulate the differential regulation of lateral and end-on attachments. Indeed, this was the case in an *in vitro* reconstitution experiment using dynamic MTs and purified Ndc80C and Dam1C — the latter containing phospho-mimetic mutants at its Aurora B phosphorylation sites [49].

In addition to Aurora B kinase, other factors also regulate kinetochore–MT interactions for error correction. The Mps1 kinase is required for error correction to achieve chromosome biorientation [50–52]. Evidence suggests that, like Aurora B kinase, Mps1 kinase weakens and disrupts aberrant kinetochore–MT interactions both *in vivo* and *in vitro* in budding yeast and human cells [50,53,54]. In yeast, Mps1 kinase is also required for the SPB duplication and SAC [55]. Through the analyses of novel *mps1* mutations and their suppressor mutations, it was suggested that the SPB duplication and biorientation are regulated by separate pathways involving Mps1 [56] while the SAC and biorientation are governed by the common Mps1 pathway [57]. For Mps1 recruitment to the kinetochore, the Mps1 N-terminus binds the backside (opposite from the MT-binding side) of Ndc80–Nuf2 CH domains and the evidence suggests that the SAC and biorientation indeed rely on this binding [58–61]. Phosphorylation of the Ndc80 N-terminus by Mps1 contributes to error correction [53], while phosphorylation of the Dam1 C-terminus by Mps1 changes kinetochore distribution in metaphase (indicative of altered kinetochore–MT interaction) [62]. However, the relevant non-phosphorylatable mutants of Ndc80 and Dam1 showed much milder defects in biorientation than an Mps1 kinase mutant [50,53,62]. Therefore, we predict that other crucial Mps1 substrates, whose phosphorylation is critical for biorientation, remain unidentified.

Another factor regulating error correction is Stu2, which is the yeast orthologue of vertebrate ch-TOG and XMAP215. Stu2 has the activity as the MT polymerase and MT nucleator [10,63,64]. However, independent of its regulation of MT dynamics, Stu2 has the activity to weaken and disrupt the kinetochore–MT interaction under low tension *in vitro* [65]. Stu2 mutants, which either fail to localize at the kinetochore or lose function specifically at the kinetochore, show biorientation defects and make cells inviable, suggesting that Stu2 at the kinetochore regulates error correction for biorientation [66,67]. It remains to be elucidated how Stu2 localized at the kinetochore promotes error correction, independently of its regulatory function on MT dynamics.

Although Aurora B and other regulators weaken the end-on attachment under low tension, this seems insufficient for efficient disruption of the end-on attachment. For example, while the kinetochore was transported by depolymerization of an end-on attached MT, the end-on attachment was rarely disrupted [15]. It was recently reported that the forces, which are generated by dynamic growth and shrinkage of kinetochore–attached MTs, facilitate disruption of the end-on attachment when the attachment is weakened under low tension (or with Dam1 phospho-mimetic mutants) [68]. In this study, the tension on the kinetochore–MT interaction was lowered by using mutants of a motor protein localizing on the interpolar MTs [68]. In physiological conditions, such forces facilitating the disruption of end-on attachment may be generated when two MTs involved in syntelic attachment (i.e. sister kinetochores that attach to the two MTs extending from the same spindle pole) concurrently show dynamic growth and shrinkage. Alternatively, even slightly different dynamics of two MTs in syntelic attachment would generate a twisting force on sister kinetochores. Such twisting force may facilitate the disruption of one of the two end-on attachments in syntelic attachment, thus resolving the aberrant attachment [69]. Once this happens, the twisting force would be relieved, and the other end-on attachment would not be lost. This would avoid a simultaneous loss of MT attachments at both sister kinetochores and prevent a chromosome from drifting away from the spindle during error correction.

## Tension stabilizes the kinetochore–microtubule interaction

Understanding the exact localization of Aurora B kinase at the centromere/kinetochore could give an important clue to its role in error correction. Aurora B localizes at the centromere/kinetochore in early mitosis and this localization continues until anaphase onset in both vertebrate cells [70] and budding yeast [71,72]. Aurora B (also known as Ipl1 in yeast) is the catalytic component of the chromosome passenger complex (CPC), which also includes INCENP, Survivin, and Borealin — or Sli15, Bir1, and Nbl1, as they are known in budding yeast [70]. The C-terminus of INCENP (Sli15) binds and activates Aurora B (Ipl1) [70,73]. The CPC is recruited to the centromere, mediated by the interaction of Survivin with Shugoshin and phosphorylated histones [74–78]. However, more recent studies showed a Survivin (Bir1)-independent CPC recruitment mechanism in budding yeast. In this mechanism, INCENP (Sli15) directly interacts with Mcm21–Ctf19 subcomplex at the inner kinetochore to facilitate the CPC recruitment [79,80]. These two mechanisms of CPC recruitment, Surivivin-dependent and -independent, function redundantly in promoting chromosome biorientation, although the Survivin-dependent mechanism is more predominant. If both mechanisms are impaired, most sister kinetochores fail to establish biorientation [79,81].

The CPC shows dynamic turnover on the centromere/kinetochore during early mitosis in human cells [82–85]. It was previously proposed that this turnover could be important for error correction as it may help Aurora B reach its distant substrates and/or facilitate Aurora B's activation on the mitotic spindle (and then to shuttle back to the centromere/kinetochore) [86,87]. Recently the dynamic turnover of CPC was also observed in budding yeast [88]. However, using an engineered recruitment of Aurora B–INCENP, it was suggested that this turnover is not required for error correction [88]. Another report indicated that the level of CPC at the kinetochore/centromere is reduced a while after the establishment of the bipolar spindle [89]. This may not regulate error correction in an unperturbed cell cycle since biorientation is rapidly established following the bipolar spindle formation [47,90], but may contribute to the maintenance of biorientation when metaphase is extended.

To establish biorientation, any aberrant kinetochore–MT interactions must be weakened and disrupted during error correction, and this process requires phosphorylation of the outer kinetochore components Dam1C and Ndc80C by Aurora B kinase, as discussed in section “Kinetochore–microtubule interactions exchange during error correction”. If the new kinetochore–MT interaction leads to the establishment of chromosome biorientation, tension is applied across sister kinetochores, which stabilizes kinetochore–MT interactions [47,91]. However, it is still not completely understood how Aurora B stops disrupting kinetochore–MT interaction when the tension is applied, i.e. how tension stabilizes kinetochore–MT interaction. To explain this tension-dependent process, several models have been proposed [69]. In this article, we focus on the following two models, for which new evidence was recently obtained.

**Figure 3.**
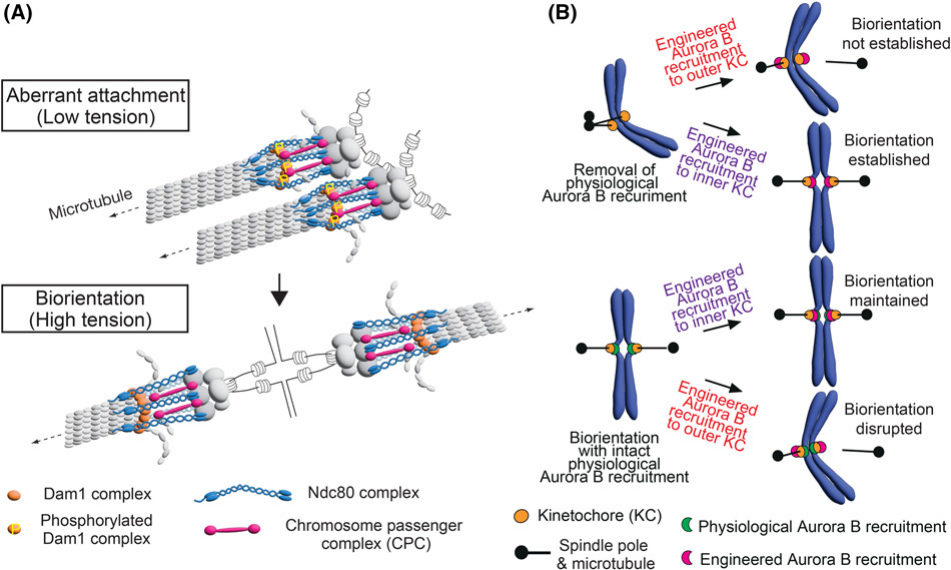
The Aurora B spatial separation model and the evidence supporting the model. (**A**) The diagram illustrates the Aurora B spatial separation model. It shows the aberrant (syntelic) kinetochore–MT attachment (top) and chromosome biorientation (bottom). To resolve the aberrant kinetochore–MT interaction, the Dam1C components must be phosphorylated by Aurora B kinase. This phosphorylation weakens kinetochore–MT interaction (top), leading to its disruption. When biorientation is established, tension is applied across sister kinetochores, which stretches the Ndc80C. This stretch spatially separates Aurora B from its outer kinetochore substrates, making kinetochore–MT interaction stable. See details in the text. (**B**) The diagram shows the outcomes of the engineered recruitment of Aurora B (Ipl1), with INCENP (Sli15), to the outer or inner kinetochore, either with or without physiological Aurora B recruitment mechanisms [88]. See details in the text.

The first is the Aurora B spatial separation model [29,92]. The Ndc80C has a long coiled-coil region and, under low tension, can bend at its kink locating in one-third from the Ndc80/Nuf2 CH domains [93,94]. According to the model, Aurora B at the centromere/inner kinetochore can reach Dam1C to phosphorylate its components under low tension (Figure 3A, top). However, when tension is applied, Ndc80C is stretched and exceeds INCENP in length, which would spatially separate Aurora B from its outer kinetochore substrates (Figure 3A, bottom). This would happen in budding yeast because the centromere and inner kinetochore, where CPC localizes, are in close proximity (<10–20 nm), whereas they are farther apart from outer kinetochore substrates of Aurora B (>60 nm) when tension is applied [43,95]. The spatial separation would lead to dephosphorylation of the outer kinetochore substrates [48,96], thus stabilizing the kinetochore–MT interaction [29,92].

The second model is the kinetochore conformational change model [97]. When tension is applied to the kinetochore–MT interaction, the kinetochore–MT interface can undergo a conformational change in such a way that the end-on attachment is stabilized. This may happen if the kinetochore conformational change either (a) results in dephosphorylation of the Aurora B substrates in Dam1C [48] or (b) overcomes the effect of Aurora B-dependent phosphorylation that is weakening the kinetochore attachment to the MT end.

The evidence for the Aurora B spatial separation model was recently obtained in budding yeast, using engineered recruitment of Aurora B–INCENP (Ipl1–Sli15) to the different kinetochore sites [88]. In the absence of physiological Aurora B–INCENP recruitment mechanism to the centromere/inner kinetochore, engineered recruitment of Aurora B–INCENP to the inner kinetochore, but not the outer kinetochore, prior to biorientation supported the subsequent establishment of biorientation [88] (Figure 3B, top). On the other hand, in the presence of physiological Aurora B–INCENP recruitment mechanism, engineered recruitment of Aurora B–INCENP to the outer kinetochore, but not the inner kinetochore, after biorientation establishment subsequently disrupted the biorientation [88] (Figure 3B, bottom). Results similar to the latter were also obtained in human cells [92,98]. These outcomes are explained by the Aurora B spatial separation model, but not by the kinetochore conformational change model alone. Nonetheless, the outcomes can also be explained if both the Aurora B spatial separation and the kinetochore conformational change mechanisms are required for stabilizing the kinetochore–MT interaction under high tension.

Meanwhile, the evidence for the kinetochore conformational change model has recently been obtained with an optical tweezer assay using purified budding yeast kinetochores *in vitro* [99]. In this assay, Aurora B was not detected in the purified kinetochore and, when kinetochore-unbound Aurora B was added to the reaction, the kinetochore–MT interaction was more stable under higher tension [99]. The result supports the kinetochore conformational change model. However, when kinetochore-unbound Aurora B was present at higher concentrations, such an effect was not observed [100]. Thus, for the kinetochore conformational change mechanism to work, the amount of Aurora B reaching its outer kinetochore substrates may need to be lowered when tension is applied. This may be facilitated by the Aurora B spatial separation mechanism. Further investigation is required to uncover molecular details involved in the kinetochore conformational change mechanism. In summary, two different assays recently supported two different mechanisms, i.e. the Aurora B spatial separation mechanism and the kinetochore conformational change mechanism. However, the results of both assays are also consistent with both mechanisms being required for tension-dependent stabilization of chromosome biorientation.

Dam1C phosphorylation by Aurora B is crucial for error correction (see section “Kinetochore–microtubule interactions exchange during error correction”). However, when biorientation is established and tension is applied across sister kinetochores, Dam1C needs to be dephosphorylated to stabilize the end-on attachment [48]. For this process, the action of Aurora B should be attenuated as described above, but phosphatases would also play important roles. In budding yeast, the PP1 phosphatase Glc7 counteracts the Aurora B (Ipl1) kinase activity [101,102]. Glc7 is recruited to the kinetochore by Spc105, Fin1 and Cin8, but none of these mechanisms are essential for biorientation or for cell viability [103–106]. This suggests either (1) Glc7 fractions, which are recruited to the kinetochore by the three proteins, are redundant in dephosphorylating Dam1C, (2) an unknown essential mechanism recruits Glc7 to the kinetochore or (3) Glc7 dephosphorylates Dam1C without localizing at the kinetochore. In addition to PP1, PP2A, which is recruited to the kinetochore by Sgo1, is also implicated in stabilizing the biorientation [107,108]. Moreover, phosphatases also play important roles in the relocation of the CPC from the centromere/kinetochore to the central spindle at the onset of anaphase [70]. When cells enter anaphase, sister chromatid cohesion is removed and tension is reduced at the kinetochore–MT interaction. The CPC removal from the centromere/kinetochore would help maintain robust kinetochore–MT interaction during anaphase. For the CPC relocation, Cdc14 phosphatase plays a major role by reverting Cdk1-dependent phosphorylation of INCENP (Sli15) in budding yeast [109]. More recently, it was also suggested that Fin1–Glc7 promotes the CPC relocation by reverting Aurora B-dependent phosphorylation of INCENP [110] and that Sgo1 sumoylation and PP2A are involved in the CPC relocation at the anaphase onset [111].

## Perspectives

- There have been several advancements in the research of error correction for chromosome biorientation over the last 5 years, using budding yeast as a model organism. We now have a better understanding of the structure of the kinetochore–MT interface, what regulators are involved in error correction, how the exchange of kinetochore–MT interaction is promoted by Aurora B, and how chromosome biorientation is stabilized when tension is applied.
- These advancements rely on the use of advanced technology such as new tools in yeast molecular genetics, advanced light microscopy, cryo-EM, highly sensitive mass spectrometry and *in vitro* reconstitution of kinetochore–MT interactions. The newly discovered mechanisms need to be tested in higher eukaryotes since many of them are expected to be conserved in evolution.
- New questions regarding the error correction mechanisms in budding yeast have also emerged, such as what is the exact process of the exchange of kinetochore–MT interaction during error corrections, how the force is applied in syntelic attachment to disrupt the weakened end-on attachment under low tension, what is the detailed molecular nature stabilizing kinetochore–MT interaction under high tension, and how Mps1, Stu2 and phosphatases regulate error correction.

## Open Access

Open access for this article was enabled by the participation of University of Dundee in an all-inclusive *Read & Publish* agreement with Portland Press and the Biochemical Society under a transformative agreement with JISC.

## Abbreviations

CH: calponin-homology
CPC: chromosome passenger complex
cryo-EM: cryo-electron microscopy
MTs: microtubules
SAC: spindle-assembly checkpoint
SPBs: spindle pole bodies
